# Suicidal Ideation in Individuals with Cerebral Palsy: A Narrative Review of Risk Factors, Clinical Implications, and Research Gaps

**DOI:** 10.3390/jcm14155587

**Published:** 2025-08-07

**Authors:** Angelo Alito, Carmela De Domenico, Carmela Settimo, Sergio Lucio Vinci, Angelo Quartarone, Francesca Cucinotta

**Affiliations:** 1 Department of Biomedical, Dental Sciences and Morphological and Functional Images, University of Messina, 98124 Messina, Italy; alitoa@unime.it; 2 I.R.C.C.S. Centro Neurolesi Bonino Pulejo, 98124 Messina, Italy; carmela.settimo@irccsme.it (C.S.); francesca.cucinotta@irccsme.it (F.C.); 3 Neuroradiology, Department of Biomedical, Dental Sciences and Morphological and Functional Images, University of Messina, 98124 Messina, Italy; sergio.vinci@unime.it

**Keywords:** cerebral palsy, suicidal ideation, mental health, adolescents with disabilities, suicide prevention, underdiagnosis, chronic conditions

## Abstract

**Background:** Cerebral palsy (CP) is a lifelong neurodevelopmental disorder characterised by motor impairment and commonly associated with comorbidities such as cognitive, communicative, and behavioural difficulties. While the physical and functional aspects of CP have been extensively studied, the mental health needs of this population remain largely underexplored, particularly concerning suicidal ideation and self-injurious behaviours. The purpose of this review is to synthesise the existing literature on suicidality in individuals with CP, explore theoretical and clinical risk factors, and identify key gaps in the current evidence base. **Methods:** A narrative literature review was conducted focusing on studies addressing suicidal ideation, self-harm, or related psychiatric outcomes in individuals with CP. Additional literature on risks and protective factors was included to support theoretical inferences and clinical interpretations. **Results:** Only a limited number of studies addressed suicidality directly in CP populations. However, several reports document elevated rates of depression, anxiety, and emotional distress, particularly among adults and individuals with higher levels of functioning. Communication barriers, chronic pain, social exclusion, and lack of accessible mental health services emerged as critical risk factors. Protective elements included strong family support, inclusive environments, and access to augmentative communication. **Conclusions:** Suicidality in individuals with CP is a neglected yet potentially serious concern. Evidence suggests underdiagnosis due to factors such as communication barriers and diagnostic overshadowing. Future research should prioritise disability-informed methodologies and validated tools for suicidal ideation, while clinicians should incorporate routine, adapted mental health screening in CP care to ensure early detection and person-centred management.

## 1. Introduction

Cerebral palsy (CP) is the most common motor disability of childhood, affecting approximately 2–3 per 1000 live births worldwide [1]. It is a group of disorders affecting movement and posture caused by non-progressive disturbances to the developing foetal or infant brain. These disorders are frequently associated with other conditions, such as intellectual impairment, communication issues, epilepsy, and musculoskeletal problems [2,3]. Although most research on CP has traditionally focused on motor outcomes and physical rehabilitation, there is a growing recognition of the psychological and social challenges experienced by people with CP throughout their lives [4].

In recent years, several studies have highlighted a significantly increased prevalence of mental health problems, in adolescents with functional limitations and disabilities, such as anxiety and behavioural disorders in individuals with intellectual disabilities [5], mental health conditions including depression and suicidal ideation in youth with CP [6,7], and complex psychopathological profiles observed in adolescents with neurodevelopmental disorders [8]. This risk appears to persist into adulthood, being more pronounced in individuals with higher functional impairments and those without cognitive disabilities. This may be due to increased self-awareness and social isolation [9,10]. However, research on more severe psychopathological phenomena, such as suicidal ideation and self-injurious behaviour, remains limited.

According to the World Health Organization (WHO) [11], suicide is a growing global public health concern and is currently among the leading causes of death in adolescents and young adults. Young people with chronic medical conditions are known to be at an increased risk of suicidal thoughts and self-harming behaviours, primarily due to the cumulative impact of physical, social, and psychological stressors. Studies of populations with epilepsy and type 1 diabetes, for example, have shown significantly higher rates of suicidal ideation (SI) than healthy peers [12,13,14]. Despite experiencing comparable or greater functional limitations, individuals with CP are rarely included in these risk profiles or suicide prevention initiatives. SI refers to thoughts of engaging in suicide-related behaviour. These thoughts can range from fleeting to detailed plans, and SI is a recognised predictor of suicide attempts and successful suicides [15]. In the general population, individuals with physical disabilities or chronic illnesses are at an increased risk of experiencing SI due to factors such as chronic pain, reduced autonomy, social exclusion, and limited access to psychological care [16,17]. However, data on suicidal ideation specifically among individuals with CP are scarce. A recent population-based study revealed that young people with CP were less likely to be diagnosed with SI or depression than their peers with other chronic conditions, such as asthma, epilepsy, and diabetes [18], raising concerns about potential under-recognition and under-diagnosis in this vulnerable group.

Considering growing concerns about the psychological well-being of people with CP and the paucity of literature addressing suicidal ideation in this population, this narrative review aims to synthesise the available evidence on suicidality. Additionally, it seeks to explore the theoretical and clinical mechanisms underlying suicidal thoughts and behaviours in this group, identify risks and protective factors, and highlight gaps in current understanding. The ultimate goal is to inform future research directions and promote more inclusive, disability-sensitive mental health surveillance and intervention approaches.

## 2. Materials and Methods

### 2.1. Data Source and Search Strategy

This narrative review was registered in the Open Science Framework (OSF) database (DOI 10.17605/OSF.IO/H2ZCQ). A comprehensive literature search was conducted using PubMed. The search was performed until 1 May 2025. The search strategy included predefined search terms and Boolean operators (AND, OR) to ensure a broader identification of relevant studies and utilised both keywords and Medical Subject Headings (MeSH) terms to enhance specificity and sensitivity. The following keywords and MeSH terms were employed: “cerebral palsy”, “suicide”, “suicidal ideation”, “self-harm”, “depression”, “anxiety”, “mental health”, and “self-injurious behaviour”. To increase the number of studies to screen, the reference lists of included studies or reviews retrieved were searched to ensure that a comprehensive list of relevant articles was considered for inclusion.

**Figure 1:**
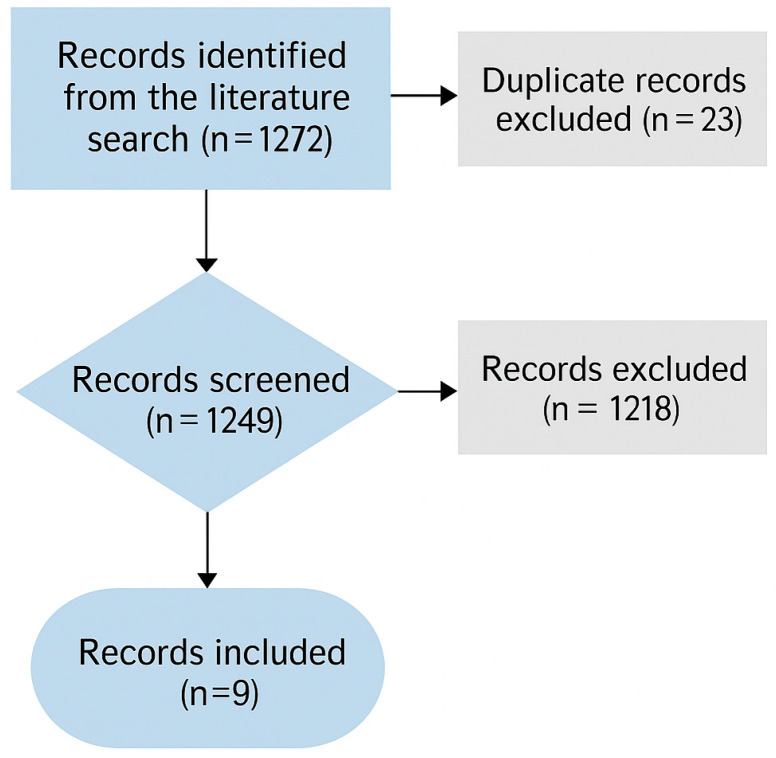
Flowchart review process.

The literature search identified 36 articles for the keyword combination “cerebral palsy” AND “suicide”, and 77 articles for “cerebral palsy” AND “non-suicidal self-injury”. Only 4 articles were retrieved using the terms “cerebral palsy” AND “suicidal ideation”. A broader search combining “cerebral palsy” AND “mental health” yielded a total of 1155 publications; among these, only 972 involved human participants. A total of 23 duplicate records were identified and removed. Following the analysis of the retrieved literature, nine articles were ultimately included. More details in Figure 1.

Inclusion criteria were based on the following: (a) CP subjects of all age; (b) articles focused on both direct evidence of suicidality and related psychiatric outcomes in this population; (c) cross-sectional and observational studies, systematic reviews, and meta-analyses published in indexed journals with peer review processes; (d) Italian, English language or studies in another language for which translation was available; and (e) availability of the full-text article. Exclusion criteria included (a) articles that did not focus on the CP sample; (b) textbooks, editorials, and letters to the editor; (c) language other than Italian, English, or without available translation; (d) unavailability of the full-text article; and (e) contents not connected to the topic of our review.

### 2.2. Selection Procedures

Study selection was conducted by two blinded authors (CDD and CS). After removing duplicates, identified references were initially screened by title and abstract. Full-text articles were then assessed for eligibility based on title, abstract, full-text content, and specificity of the topic. In case of disagreement, a third author (AA) was consulted to reach a consensus. When overlapping studies were identified, the largest study was included.

## 3. Overview of the Literature

The growing attention to mental health in neurodisability research reflects a significant shift from a traditional biomedical focus on motor deficits to a more comprehensive understanding of psychosocial well-being across the lifespan. Despite these advances, the emotional and psychological challenges faced by individuals with CP remain substantially understudied. New evidence highlights the prevalence of emotional dysregulation, depressive symptoms, and anxiety in this population, all of which may contribute to an elevated risk of suicidality [19,20]. Table 1 summarises the main characteristics of the studies included in this review.

### 3.1. Prevalence of Mental Health Disorders in Cerebral Palsy

Several studies have consistently demonstrated that individuals with CP are at a significantly elevated risk of experiencing mental health problems throughout their lifespan. For instance, a large cross-sectional study in the United Kingdom identified that 57% of children with CP had clinically significant emotional or behavioural difficulties, with hyperactivity, peer problems, and emotional symptoms being the most prevalent [22]. Similar findings have been reported in adolescence and adulthood, with a recent Australian study documenting moderate to extremely severe symptoms of anxiety and depression in 60% and 33% of adults with CP, respectively [19]. These elevated rates appear to be independent of the level of motor impairment, cognitive function, or sociodemographic characteristics, suggesting that the experience of living with CP itself may confer a unique psychological vulnerability [6].

This vulnerability is also seen in adolescents and young adults with other childhood-onset physical disabilities, who exhibit disproportionately high rates of sadness, hopelessness, and suicidal behaviours compared to their peers [20]. The developmental period of adolescence, marked by increased social comparison, identity formation, and the transition to adult care systems, may amplify these risks in individuals with CP, who often experience reduced autonomy, social exclusion, and environmental barriers to participation [20,24].

### 3.2. Suicidal Ideation and Risk Factors in CP

Despite the high prevalence of mental health problems, there is a paucity of literature specifically addressing SI and risk behaviour in individuals with CP. Nevertheless, available evidence suggests that suicide risk may be underdiagnosed in this group, in part due to communication barriers, atypical behavioural presentations, and limited routine mental health screening [23,25]. A population-based study using hospital records found that individuals with CP were less likely to receive a formal diagnosis of depression, and the recognition of the depressive symptoms and red flags of suicide risk is reduced compared with other chronic illnesses, despite exhibiting comparable functional impairments [25]. This under-recognition may reflect systemic gaps in diagnostic procedures rather than a true lower prevalence.

Several psychosocial and neurobiological factors may contribute to heightened suicide risk in individuals with CP. First, emotion regulation difficulties, such as impulsivity, rumination, and poor distress tolerance, have been identified as significant correlates of depression and anxiety in adults with CP [19]. Emotional dysregulation has also been associated with suicidal thinking in broader psychiatric populations, and its presence in CP could be partly explained by the presence of impairment of fronto-limbic pathways involved in affective processing. This is particularly relevant in dyskinetic CP, where basal ganglia damage may alter affective reactivity and regulation [19].

Furthermore, social isolation and perceived burdensomeness may act as powerful psychological stressors in people with CP, especially in those with preserved cognitive abilities who are acutely aware of their limitations and social positioning [23]. These factors are central to the Interpersonal Theory of Suicide [26], which suggests that the desire to die stems from a sense of not belonging and the belief that one is a burden to others, exacerbated by the ability to cause oneself harm. Individuals with CP often experience chronic dependence on caregivers, reduced employment opportunities, and limited romantic relationships—factors that can lead to a diminished sense of self-worth and autonomy [20].

In addition, physical pain, fatigue, and sleep disturbances, common in individuals with CP, are recognised predictors of suicidal ideation in chronic illness populations [18]. Pain has been shown to mediate the relationship between disability and depressive symptoms [23]. While fatigue and poor mental health outcomes have been shown to improve with targeted interventions [24], few psychosocial rehabilitation programmes for CP currently integrate suicide risk assessments or emotional well-being modules.

### 3.3. Structural and Systemic Barriers to Detection and Care

The identification and management of suicidality in individuals with CP is further complicated by systemic and structural issues. As highlighted by Lal et al. [20], youth with childhood-onset physical disabilities often receive care in rehabilitation settings where the focus is predominantly physical, and providers may lack training or confidence in addressing psychiatric issues. Furthermore, the transition from paediatric to adult services is frequently fragmented, with a loss of continuity that can exacerbate mental health vulnerabilities during a critical developmental window [27].

Additionally, many standardised psychiatric assessment tools rely heavily on verbal communication or self-report measures. These may not be appropriate for individuals with significant motor, speech, or intellectual impairments [28]. The lack of validated instruments for these populations creates a methodological blind spot, potentially contributing to underreporting and underdiagnosis [25]. Studies have called for the adaptation of existing mental health screening protocols to accommodate alternative communication modalities and for the involvement of multidisciplinary teams in assessment [20,21].

Stigma also represents a huge barrier [29]. Stigmas related to both disability and mental illness may discourage individuals with CP and their families from seeking psychological help, and clinicians may inadvertently minimise emotional distress by attributing behavioural concerns to cognitive limitations or neurological damage [19,22]. This diagnostic overshadowing not only delays intervention but also reinforces a culture of therapeutic nihilism regarding emotional and behavioural health in neurodevelopmental disability. A recent systematic review, which comprehensively synthesised all available treatment studies for CP, highlights a critical gap in the literature: the absence of specific intervention programs targeting psychiatric disorders within this population [30].

### 3.4. Protective Factors and Opportunities for Intervention

Despite these challenges, several protective factors have been identified. Social support, particularly from family, peers, and inclusive community programmes, can buffer against the psychological impacts of disability. Interventions promoting self-efficacy, resilience, and adaptive emotion regulation have shown promise in improving mood and social outcomes in adolescents with CP [24]. For example, a lifestyle-based intervention targeting physical activity, coping skills, and peer interactions yielded significant improvements in perceived social support and mental health outcomes [24].

Education and vocational inclusion may also act as protective mechanisms by enhancing autonomy, purpose, and financial security. Programmes supporting school-to-work transitions and community participation have been proposed as crucial components of a comprehensive suicide prevention strategy in young people with disabilities [20].

Moreover, recent calls for integrated models of care, incorporating mental health, social work, and rehabilitation services, reflect a growing consensus on the need for disability-sensitive mental health frameworks [20]. Such models could include routine mental health screening, staff training on psychiatric comorbidities in CP, and streamlined referral pathways to psychological services. The development of adapted psychotherapeutic approaches, such as augmentative communication-supported Cognitive Behavioural Therapy (CBT) or caregiver-mediated interventions, represents a further area for innovation [22].

### 3.5. Implications and Practical Recommendations

Several practical implications for clinical practice, research, and policy emerge from the findings. Firstly, screening tools must be adapted to the communication and cognitive needs of individuals with CP, using simplified language, visual aids, or augmentative and alternative communication (AAC) systems. Such strategies have been shown to be effective in identifying emotional distress in individuals with intellectual disabilities [10,31].

Secondly, mental health support should be integrated into routine CP care. Despite evidence that mental health professionals and school-based inclusion programmes can reduce isolation and emotional distress [32,33], multidisciplinary services often fail to consider psychological wellbeing.

Third, clinician education and practice must address diagnostic oversight and equip providers to recognise and manage mental health symptoms in people with complex needs [32]. National training frameworks should embed this competence across professional roles.

Fourth, future research should include longitudinal and co-produced studies to capture the lived experience of mental health across the lifespan in CP, with tools tailored for diverse functional profiles.

Finally, these recommendations should inform disability-inclusive policy. National suicide prevention strategies and mental health frameworks must explicitly include individuals with CP, in alignment with the UN Convention on the Rights of Persons with Disabilities [34] and the WHO Mental Health Action Plan [35].

However, achieving meaningful change requires a clear commitment from clinicians, researchers and policymakers to develop and implement practical, inclusive strategies that address the specific needs of individuals with CP. Early and adapted prevention strategies, especially for young people at increased risk, are essential to reduce long-term psychiatric morbidity, as emphasised in broader mental health policy frameworks [33].

### 3.6. Research Gaps and Future Directions

Despite growing awareness, large-scale epidemiological studies on suicidality in CP remain sparse. There is a pressing need for longitudinal research to elucidate developmental trajectories of mental health in CP and to determine the timing and persistence of suicide risk factors. Furthermore, future studies should explore the presence of emotional dysregulation in this population, investigate its neurobiological basis, and examine the differential effects of CP subtype, severity, and comorbidities on psychiatric outcomes.

Parents of children with CP frequently experience significant psychological distress, often driven by affiliate stigma and insufficient social support [36]. Recent evidence also indicates elevated rates of anxiety and depression among both parents and grandparents, highlighting the multigenerational impact of caregiving in contexts lacking adequate resources [37].

Despite the increasing recognition of parental stress and caregiver burden in families of children with CP, relatively few studies have directly explored quality of life (QoL) and mental health problems from the perspective of children and adolescents with CP themselves [38]. The physical quality of life of people with CP is often more impaired. However, the connection between CP and psychosocial quality of life is less obvious. In particular, discrepancies between parent-proxy and child self-reports underscore the importance of taking a multi-informant approach to assessing QoL in this population. Such differences may reflect different perceptions of well-being, coping strategies, and contextual factors [39]. From a rehabilitation perspective, QoL is a critical health outcome, and integrating the child’s accounts and parents’ perspectives provides a more nuanced understanding of the child’s lived experience. It therefore appears necessary to adopt a dual-informant strategy as it can provide essential information to identify domain-specific challenges that are uniquely salient to the child or his or her caregivers, ultimately guiding more individualised and effective interventions [40].

Efforts must also be made to include the voices of individuals with CP in research design, particularly regarding their experiences of emotional distress, suicidality, and engagement with mental health services. Qualitative and mixed-methods approaches may be particularly suited to this purpose, especially in capturing nuanced experiences of internalised stigma, relational stress, and healthcare interactions.

Finally, suicide prevention initiatives at the policy level must explicitly include people with neurodevelopmental disabilities in their target populations. Public health campaigns, school-based mental health promotion, and national suicide registries should ensure appropriate categorisation and monitoring of suicide risk among individuals with CP to address existing disparities in care and prevention.

This narrative review has several limitations, including an absence of systematic search and appraisal procedures and the potential omission of relevant studies. A limitation of our methodology is the use of PubMed as the sole database. While PubMed provides extensive biomedical coverage, this may have limited the retrieval of relevant studies available in other sources. We recognise this as a potential source of bias and suggest that future work adopt a broader database strategy. Additionally, the available literature is characterised by variability in study designs, participant characteristics, and assessment tools, which may limit the comparability and generalisability of the findings. The review structure was developed with attention to commonly recognised standards for narrative reviews, including clarity of objectives, transparency in the literature search, and coherence in the synthesis of evidence. These elements were prioritised to improve the review’s transparency and clinical relevance, while acknowledging the limitations inherent to the available literature. Finally, although the current evidence base remains limited, the convergence of findings highlights the urgency of addressing mental health vulnerabilities in individuals with conditions such as cerebral palsy. Ensuring access to appropriate care is not only a clinical responsibility, but also a public health and ethical priority.

## 4. Conclusions

Mental health issues, including suicidal thoughts, are common and often overlooked in people with CP. This review has highlighted how diagnostic overshadowing, communication barriers, and structural exclusion can lead to systematic gaps in care. These omissions are not just clinical oversights; they reflect a broader failure to recognise the psychological needs of a marginalised group.

Moving forward requires more than just clinical awareness. It requires a commitment to inclusive research, the co-design of adapted assessment tools, and the integration of mental health services into disability and rehabilitation programs. Above all, mental health must be treated as a fundamental right, not an optional adjunct, for individuals with CP.

Ensuring this population is no longer invisible within mental health systems is not only a matter of best practice, but also of equity.

## Figures and Tables

**Table 1:** Characteristics of included studies.

**ID**	Author	Year	Country	Sample	Sample Age Mean ± SD (Range)	Sex (M:F)	Ethnicity	Outcome Measures	Main Findings	CP-Related Associations
#1	Bhatnagar et al. [18]	2024	United States (Midwest), North America	216,794	4.3 ± 5.1 years (0–21 years)	55% M	76,2% White, 94,7% non-Hispanic	CCSR	Higher anxiety, ADHD, and conduct/impulsivity; lower rates of depression and suicidal ideation (potential underdiagnosis)	Males showed higher ADHD and conduct problems; GMFCS levels III–V linked to more psychiatric diagnoses; Black and other ethnicities had higher rates of OCD, mood, and trauma/stress disorders.
#2	Bjorgaas et al. [6]	2013	Norway, Europe	47	8.5 years (4–12 years)	NR	NR	Kiddie-SADS SDQ	67% above 90th percentile (SDQ); 57% diagnosed with ≥1 psychiatric disorder (Kiddie-SADS); SDQ sensitive but not specific	Multiple co-occurring symptoms; peer relationship problems very common
#3	Clark et al. [21]	2000	United Kingdom (London), Europe	47	NR (4–12 years)	NR	NR	NR	High rates of bulbar problems (80%), drooling (86%), otitis media (60%), reflux/nutrition/aspiration issues (40%); mild tetraplegia in 91%, learning difficulties in 81%, neuropsychiatric problems in 41%, epilepsy in 28%; mean age at diagnosis 6 years; 32% had perisylvian polymicrogyria on neuroimaging.	Multisystem condition; overlapping phenotype with bilateral perisylvian syndrome; classified as a form of CP
#4	Honan et al. [19]	2023	Australia (Sydney), Oceania	42	31.5 ± 13.5 years (NR)	NR	NR	DASS-21, DERS	High emotional difficulties: 33% showed moderate–severe depression and 60% moderate–severe anxiety; poor emotion regulation (DERS) closely linked to higher depression, anxiety, and stress.	Problematic emotion regulation associated with increased depression, anxiety, and stress
#5	Lal et al. [20]	2022	Canada, North America	NA	NR (13–24 years)	NR	NR	NA	Most common mental health issues: depression/mood (73% of studies), anxiety (39%), and social/behavioural difficulties (33%)	Parent reports indicate higher mental health problems in CP compared to controls
#6	Parkes et al. [22]	2008	Europe	818	NR (8–12 years)	NR	NR	SDQ	Higher odds of emotional and behavioural difficulties in children with CP versus typically developing peers	Physical and environmental contributors to psychological distress in CP
#7	Power et al. [23]	2019	Bangladesh, Asia	327	15.1 ± 1.6 years (CP); 14.9 ± 1.6 years (controls) (NR)	31.2% F (CP); 31.8% (controls)	NR	CPQoL-Teens, KIDSCREEN-27, SDQ	Adolescents with CP had poorer HRQoL and higher SDQ total difficulties than controls; odds of a “probable” SDQ total score were 7.8 times higher (self) and 12.0 times higher (proxy).	CP in rural areas associated with lower quality of life, compromised mental health, and impact of scarce resources
#8	Slaman et al. [24]	2015	Netherlands, Europe	57	NR (16–24 years)	NR	NR	FSS, CIS-f, HRQoL, GMFM, SSEBS, IMI, GSE	Reduced fatigue and pain; improved mental health and social support; effects partly mediated by increased physical activity and fitness.	Improvements partly mediated by changes in physical activity and fitness; social support changes minimally explained by these, suggesting independent psychosocial factors
#9	Smith et al. [25]	2019	United Kingdom (London), Europe	6820	33.3 ± 15.5 years (NR)	46.8% F	NR	NR	Increased risk of depression and anxiety	Higher risk in CP without ID; no significant increase in CP with ID

Legend: ADHD: Attention Deficit Hyperactivity Disorder; CCSR: Clinical Classifications Software Refined; CIS-f: fatigue subscale of the Checklist Individual Strength; CP: Cerebral Palsy; CPQoL-Teens: Quality of Life-Teens; DASS-21: Depression Anxiety and Stress Scale-21, DERS: Difficulties in Emotion Regulation Scale; FSS: Fatigue Severity Scale; GMFCS: Gross Motor Function Classification System; GMFM: Gross Motor Function Measure; GSE: General Self-Efficacy scale; HRQoL: Health-Related Quality of Life; ID: Intellectual Disability; IMI: Intrinsic Motivation Inventory; NA: Not Applicable; NR: Not Reported; OCD: Obsessive-Compulsive Disorder; SD: Standard Deviation; SDQ: Strengths and Difficulties Questionnaire; SSEBS: Social Support for Exercise Behaviour Scale.

## Data Availability

The data presented in this study are available on request from the corresponding author.

## Abbreviations

The following abbreviations are used in this manuscript:

CP	Cerebral Palsy
WHO	World Health Organization
SI	Suicidal Ideation
OSF	Open Science Framework
MeSH	Medical Subject Headings
CBT	Cognitive Behavioural Therapy
AAC	Augmentative and Alternative Communication
QoL	Quality of Life
